# Opioid addiction and withdrawal differentially drive long-term depression of inhibitory synaptic transmission in the hippocampus

**DOI:** 10.1038/srep09666

**Published:** 2015-05-05

**Authors:** Huili Han, Zhifang Dong, Yunfang Jia, Rongrong Mao, Qixin Zhou, Yuexiong Yang, Liping Wang, Lin Xu, Jun Cao

**Affiliations:** 1 Ministry of Education Key Laboratory of Child Development and Disorders, Children’s Hospital of Chongqing Medical University, Chongqing 400014, China; 2 Chongqing Key Laboratory of Translational Medical Research in Cognitive Development and Learning and Memory Disorders, Children’s Hospital of Chongqing Medical University, Chongqing 400014, China; 3 Key Laboratory of Animal Models and Human Disease Mechanisms, and KIZ/CUHK Joint Laboratory of Bioresources and Molecular Research in Common Disease, and Laboratory of Learning and Memory, Kunming Institute of Zoology, Chinese Academy of Sciences, Kunming 650223, China; 4 Kunming College of Life Science, University of Chinese Academy of Sciences, Beijing 100049, China

## Abstract

Addictive behavior is increasingly accepted as a drug-associated pathological memory in which the hippocampus is profoundly engaged. It has been well documented that adaptations of synaptic plasticity of excitatory transmission in the hippocampus may contribute to opioid addiction. However, it remains unknown whether and how adaptive changes of synaptic plasticity of inhibitory transmission in the hippocampus occurs during opioid abuse. Here, we reported that a single in vivo morphine exposure (SM) did not affect inhibitory long-term depression (I-LTD) in the hippocampus, compared with saline control; while repeated morphine exposure (RM) abolished this I-LTD. Interestingly, opioid withdrawal for 3-5 days after repeated (RMW), but not a single morphine exposure (SMW), largely enhanced I-LTD. More importantly, the I-LTD in single morphine treatment is dependent on presynaptic mechanism since it can be blocked by AM251, a selective cannabinoid receptor 1 antagonist. While the large I-LTD in RMW group is dependent on combinatorial presynaptic and postsynaptic mechanisms since it can be blocked by co-application of AM251 and L-type calcium channel blocker LaCl_3_. Thus, these results demonstrate that opioid use and withdrawal drive the dynamics of presynaptic and postsynaptic I-LTD expression in the hippocampus that may contribute to the persistent behavioral changes during opioid abuse.

The persistence of drug addiction is characterized by the reoccurrence of drug-seeking and -taking behaviors triggered by drug-related cues even years after withdrawal. In recent years, a growing body of evidence has shown that memory mechanisms are likely engaged in this pathological process^1^,^2^,^3^,^4^. The hippocampus is well known to be critical in the formation of several types of long-term memory, including addictive memory. For example, our previous report has shown that blocking hippocampal glucocorticoid receptors prevents morphine-induced conditioned place preference behavior^5^. Further imaging study demonstrates that opioid exposure increases activation of the nucleus accumbens (NAc) and hippocampus in the drug-naïve human subjects^6^. In addition, cocaine-associated memory is retrieved by electric stimulation to the hippocampal-NAc pathway and thus triggers relapse in rats even long after cocaine withdrawal^7^,^8^,^9^.

Activity-dependent synaptic plasticity, particularly long-term potentiation (LTP) and long-term depression (LTD), has been proposed as a cellular mechanism underlying learning and memory^10^,^11^. Single morphine exposure induces LTP-like modification and facilitates the induction of LTD^12^, but repeated opioid exposure gradually abolishes the induction of LTP^13^ and 4-day opioid withdrawal after repeated morphine exposure drives an enhanced LTP in the hippocampus *in vivo*^14^. Similar adaptations of LTP are also found in the hippocampal-NAc pathway *in vivo*^15^. Together, these reports support that synaptic plasticity in the hippocampus is involved in drug-associated pathological learning and memory process. However, all these findings focus on the potential role of synaptic plasticity at the excitatory glutamatergic synapses in drug addiction. Correlations between drug-related memory and synaptic plasticity at the inhibitory synapses in the hippocampus have not been extensively investigated.

It has been reported that long-term potentiation (I-LTP) or long-term depression (I-LTD) of inhibitory synaptic transmission can modify LTP or LTD of excitatory synaptic transmission^16^. Activation of opioid receptors may cause hyperpolarization of inhibitory neurons, leading to disinhibition of excitatory neurons in the hippocampus^17^ or dopamine neurons in the VTA^18^. More recent reports demonstrate that single *in vivo* opioid exposure blocks I-LTP of dopamine neurons in the VTA^19^, which is mediated through the activation of μ-opioid receptors^20^. Repeated cocaine exposure induces I-LTD-like modification in VTA dopamine neurons^21^, while enhances inhibitory synaptic transmission in VTA GABA neurons^22^. Furthermore, kappa opioid receptors antagonist can reverse the stress-induced block of I-LTP and reinstatement of cocaine-seeking behavior^23^. Although these reports suggest that inhibitory synaptic plasticity may be involved in drug addiction, it is unclear whether and how I-LTD changes in the hippocampus during opioid addiction and withdrawal. In the present study, we recorded inhibitory postsynaptic currents (IPSCs) in CA1 pyramidal neurons by stimulation of Shaffer collateral/commissural pathways in rat hippocampal slices by using whole-cell voltage-clamp techniques, and examined the changes of I-LTD during single or repeated morphine exposure and withdrawal.

## Results

### Repeated *in vivo* morphine exposure abolished I-LTD in the hippocampus

**Figure 1:**
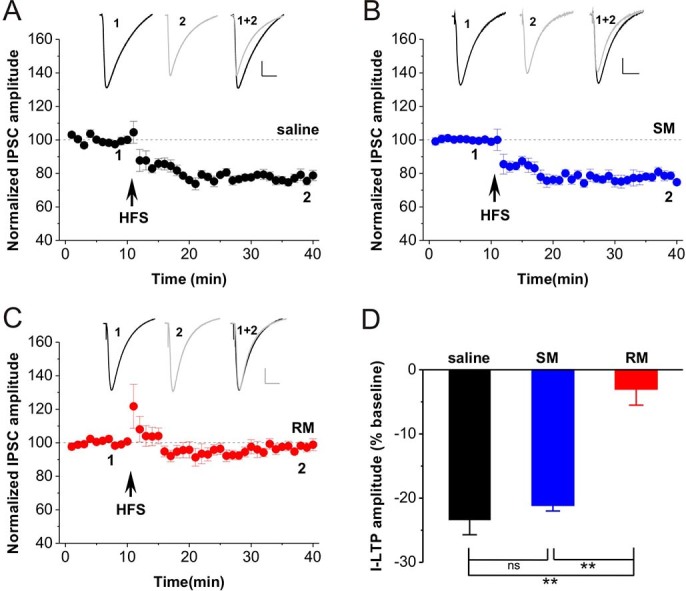
Repeated morphine exposure abolished I-LTD. **(A)** High-frequency stimulation (HFS) combined with postsynaptic depolarization induced a reliable I-LTD in saline slices. **(B)** HFS induced I-LTD in SM slices. **(C)** HFS failed to induce I-LTD in RM slices. **(D)** The bar graph summarized the average percentage change of IPSC amplitude before and 30 min after HFS. **p < 0.01, post hoc Turkey’s test after ANOVA (F _(2, 19)_ = 31.222; p < 0.001). Representative traces from corresponding hippocampal slices are shown above (Scale bar: horizontal = 50 ms, vertical = 50 pA). SM: single in vivo morphine exposure; RM: repeated in vivo morphine exposure.

Previous study has shown that high-frequency stimulation enables to induce I-LTD at hippocampal CA1 inhibitory synapses^24^. Consistent with this result, we found that HFS induced a reliable I-LTD in slices taken from rats subjected to single saline treatment (saline, n = 7, 76.6 ± 2.2%, p < 0.001 vs. baseline; Fig. 1A and 1D). Similarly, single in vivo morphine exposure had no effect on I-LTD induction since HFS induced a similar magnitude of I-LTD in slices taken from rats subjected to single morphine treatment (SM, n = 8, 78.9 ± 0.9%, p < 0.001 vs. baseline, p = 0.682 vs. saline; Fig. 1B and 1D). Interestingly, repeated in vivo morphine exposure for 12 days abolished hippocampal I-LTD induced by HFS (RM, n = 7, 97.0 ± 2.5%, *p* = 0.140 vs. baseline, p < 0.001 vs. saline, p < 0.001 vs. SM; Fig. 1C and 1D). These results suggest that repeated rather than single in vivo morphine exposure dramatically inhibits I-LTD induction in the hippocampal CA1 pyramidal neurons.

### Opioid withdrawal after repeated *in vivo* morphine exposure dramatically enhanced I-LTD

**Figure 2:**
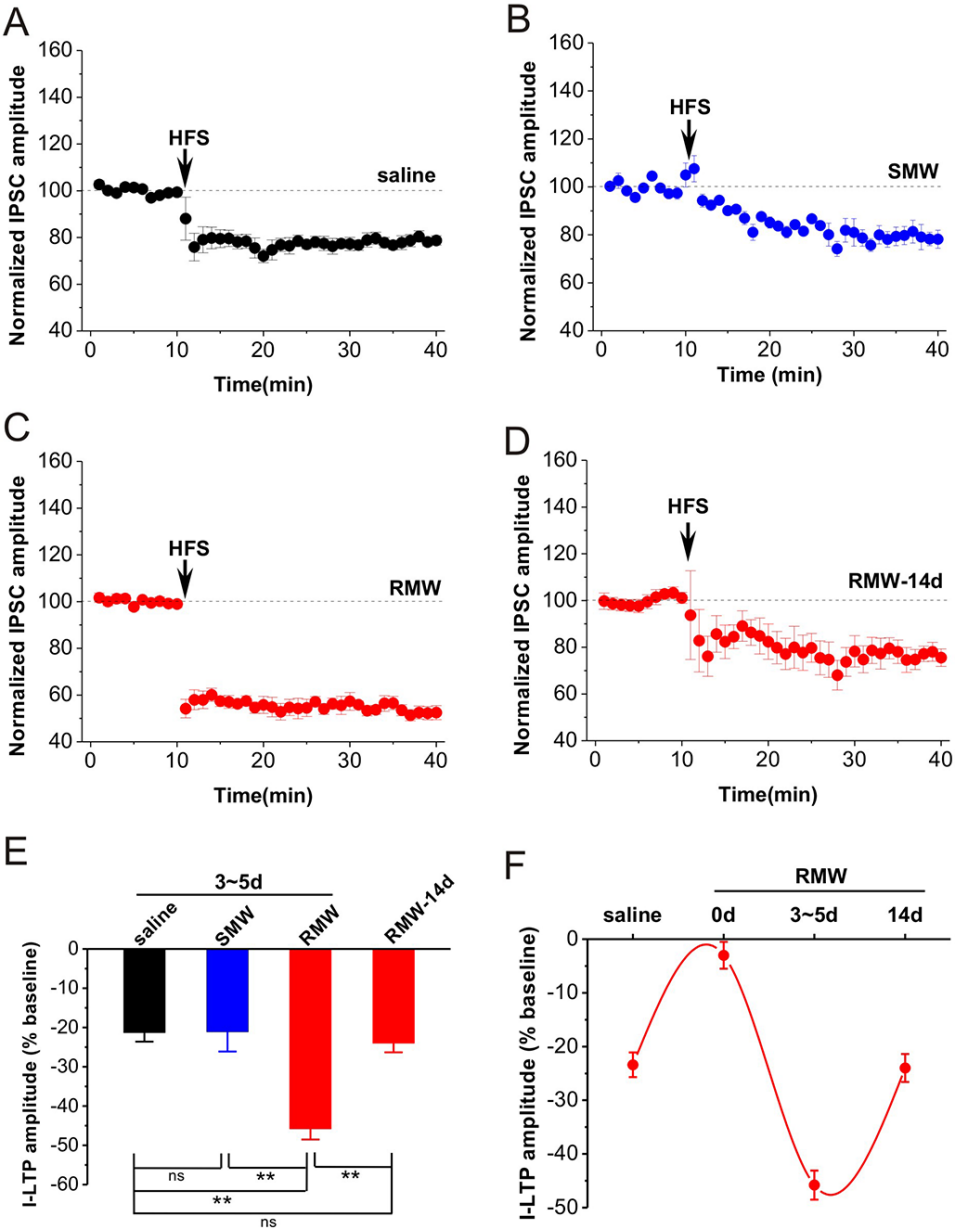
Opioid withdrawal after repeated morphine exposure enhanced I-LTD. **(A)** HFS induced a reliable I-LTD in saline group. **(B)** HFS induced a similar I-LTD in SMW slices. **(C, D)** Morphine withdrawal for 3-5 days (RMW), but not for 14 days (RMW-14d), enables HFS induced an enhanced I-LTD. **(E)** The bar graph summarized the average percentage change of IPSC amplitude before and 30 min after HFS. **(F)** The alterations of I-LTD during opioid withdrawal after repeated morphine exposure. **p < 0.01, post hoc Turkey's test after ANOVA (F_(3, 24)_ = 217.920; p < 0.001). SMW: withdrawal for 3-5 days after single in vivo morphine exposure; RMW: withdrawal for 3-5 days after repeated in vivo morphine exposure; RMW-14d: withdrawal for 14 days after repeated in vivo morphine exposure.

Next, we further examined whether opioid withdrawal affected I-LTD induction in the hippocampus. Rats were treated with single or repeated morphine and subsequently subjected to withdrawal for 3-5 days. The results showed that HFS induced a reliable I-LTD in slices taken from saline-treated rats (saline, n = 10, 78.7 ± 2.2%, p < 0.001 vs. baseline; Fig. 2A, 2E and 2F). Similarly, withdrawal after single in vivo morphine exposure had no effect on I-LTD induction since HFS induced a similar magnitude of I-LTD in slices taken from rats subjected to withdrawal after single morphine treatment (SMW, n = 3, 78.9 ± 2.7%, p < 0.001 vs. baseline; p = 0.998 vs. saline; Fig. 2B and 2E). Remarkably, withdrawal after repeated in vivo morphine exposure for 12 days dramatically enhanced hippocampal I-LTD induced by HFS (RMW, n = 7, 52.4 ± 2.6, p < 0.001 vs. baseline, p < 0.001 vs. saline, p < 0.001 vs. SMW; Fig. 2C, 2E and 2F). This enhancement may be attributed to the lower threshold for I-LTD induction because the basal inhibitory synaptic transmission significantly enhanced in RMW group, as reflected by increased miniature IPSC (mIPSC) frequency (supplementary Fig. 2A and 2B) and unchanged mIPSC amplitude (supplementary Fig. 2A and 2C) as well as I-V curve (supplementary Fig. 1). Since our previous reports have shown that prolonged morphine withdrawal restored the LTP and LTD of excitatory synaptic transmission to control level, we next tested the I-LTD in rats subjected to 14-day morphine withdrawal after repeated in vivo morphine exposure. As shown in Figure 2D, the I-LTD was suppressed in rats subjected to withdrawal for 14 days (RMW-14d, n = 8, 76.0 ± 2.6, p < 0.001 vs. baseline, p = 0.864 vs. saline, p < 0.001 vs. RMW; Fig. 2D-2F), to a level similar to that found at saline group. These results suggest that opioid withdrawal after repeated rather than single in vivo morphine exposure dramatically enhances I-LTD induction in the hippocampal CA1 pyramidal neurons.

### Withdrawal followed by repeated in vivo morphine exposure enabled a combinatorial plasticity containing both CB1-mediated presynaptic and LTCC-mediated postsynaptic I-LTD components

**Figure 3:**
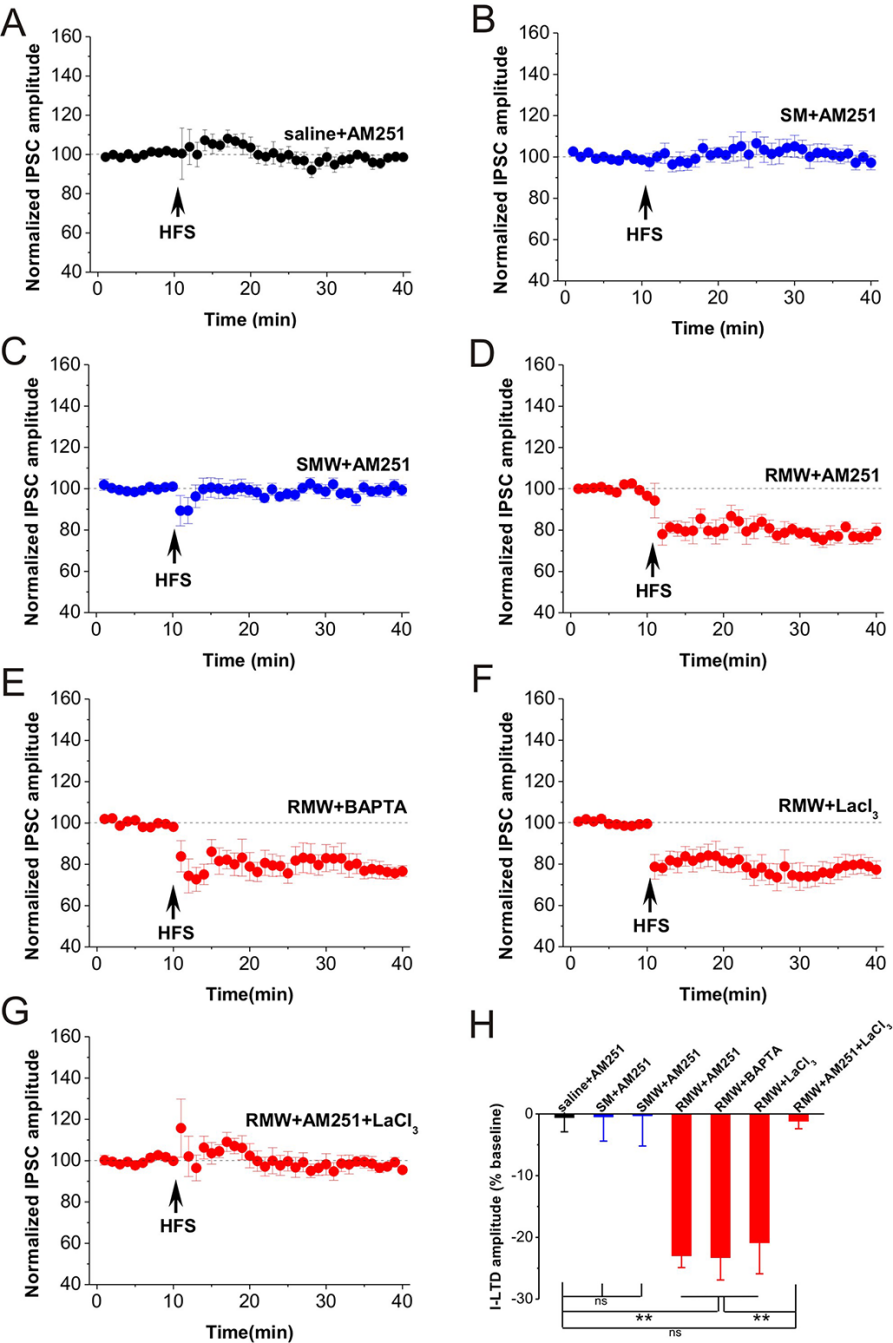
I-LTD driven by opioid withdrawal is dependent on both CB1R and LTCC. **(A)** AM251, selective CB1 antagonist AM251 (2 μM, final concentration), completely blocked the induction of I-LTD in saline slices. **(B-C)** AM251 also completely blocked the induction of I-LTD in SM and SMW slices. **(D)** AM251 only partially blocked I-LTD in RMW slices, indicating that a part of this I-LTD was depended on CB1. **(E)** The calcium chelator BAPTA (20 mM in pipette solution) partially blocked I-LTD in RMW slices. **(F)** I-LTD in RMW group was also partially blocked by L-type Ca2+ channel blocker lanthanum chloride (LaCl3; 20 µM). **(G)** Bath application of AM251 and LaCl3 completely blocked I-LTD in RMW slices. **(H)** The bar graph summarized the average percentage change of IPSC amplitude before and 30 min after HFS. **p < 0.01, post hoc Turkey’s test after ANOVA (F _(6, 46)_ = 12.450; p < 0.001).

Cannabinoid receptor 1 (CB1) is distributed in the presynaptic terminals of inhibitory synapses onto pyramidal neurons. A previous report demonstrates that CB1-mediated retrograde signaling is important for the induction of I-LTD in CA1 pyramidal neuron^24^ and is associated with stress-induced behaviors^25^. Thus, we first wanted to confirm whether the hippocampal I-LTD was dependent on CB1. The selective CB1 antagonist AM251 (2 μM) was applied into bath solution for 20 min. A 10-min baseline was recorded and then HFS was applied to induce I-LTD. AM251 completely blocked the induction of I-LTD in slices taken from rats subjected to saline treatment (saline+AM251, n = 13, 99.4 ± 2.3%, *p* = 0.697 vs. baseline; Fig. 3A and 3H). Similarly, AM251 also blocked the induction of I-LTD in slices taken from rats subjected to single morphine treatment with or without withdrawal (SM+AM251, n = 6, 99.5 ± 3.9%, *p* = 0.452 vs. baseline; SMW+AM251, n = 3, 99.7 ± 4.90%, *p* = 0.481 vs. baseline; Fig. 3C, 3D and 3H). Consistent with previous report^24^, these results indicate that hippocampal I-LTD in rats subjected to saline or single morphine treatment is dependent on presynaptic CB1.

However, AM251 only partially blocked I-LTD in slices of rats subjected to withdrawal for 3-5 days followed by repeated in vivo morphine exposure for 12 days (RMW+AM251, n = 7, 78.3 ± 2.8%, *p* < 0.001 vs. baseline; *p* < 0.001 vs. saline+AM251; Fig. 3D and 3H), to an extent similar to that of the saline-treated group (*p* = 0.294 vs. saline in Fig. 1A). These results illustrated that repeated *in vivo* opioid exposure abolished CB1-dependent presynaptic I-LTD but subsequent withdrawal restored it. However, the residual component of I-LTD with the presence of AM251 is independent on presynaptic CB1.

Since inhibitory synaptic transmission may also depend on presynaptic and postsynaptic intracellular Ca^2+^ levels or voltage-dependent Ca^2+^ channels^26^,^27^, we further examined whether the residual CB1-independent I-LTD shared these mechanisms. To address this question, we first loaded the recording pipette with the Ca^2+ ^chelator BAPTA (20 mM). Interestingly, the large I-LTD in RMW group was also blocked by BAPTA only partially (RMW+BAPTA, n = 8, 76.7 ± 3.4%, *p* < 0.001 vs. baseline; *p* <0.001 vs. saline+AM251; Fig. 3E and 3H), suggesting that the enlarged I-LTD may partially depend on postsynaptic Ca^2+^ influx. Since NMDA and AMPA/kainate receptors-mediated excitatory postsynaptic currents (EPSCs) were blocked in these studies to isolate IPSCs, the Ca^2+ ^influx could be mediated by voltage-dependent calcium channels (VDCC) such as L-type calcium channel (LTCC), which are associated with reinstatement of nicotine addictive behaviors^28^ and stressful events^29^. Thus, we bath applied the L-type Ca^2+^ channel blocker LaCl_3_ (20 μM), and as expected, this large I-LTD was partially blocked (RMW+LaCl_3_, n = 8, 79.1 ± 4.2%, *p* < 0.001 vs. baseline; *p* = 0.01 vs. saline+AM251; Fig. 3F and 3H), to an extent similar as that of RMW+BAPTA group (*p* = 0.601 vs. RMW+BAPTA). These results also suggest that the CB1-dependent presynaptic I-LTD does not depend on activation of postsynaptic L-type Ca^2+^ channel. Finally, we wanted to determine whether this large I-LTD depended on a combinatorial mechanism with both CB1- and LTCC-mediated components. The result showed that the large I-LTD was completely blocked by applying AM251 and LaCl_3_ in bath solution in slices of rats subjected to withdrawal followed by repeated in vivo morphine exposure (RMW+AM251+ LaCl_3_, n = 8, 97.7 ± 2.3%, *p* = 0.147 vs. baseline; Fig. 3G and 3H).

Together, these findings demonstrate for the first time that a combinatorial plasticity containing both CB1R- mediated presynaptic and LTCC-mediated postsynaptic components occurs during opioid addiction and withdrawal.

## Discussion

The main finding of this study is that repeated in vivo morphine exposure for 12 days abolishes I-LTD induced by HFS in hippocampal slices, while subsequent withdrawal for 3-5 days enables HFS to induce an enhanced I-LTD. More importantly, our further experiments indicate that I-LTD in slices of rats subjected to a single in vivo morphine exposure or subsequent withdrawal is dependent on presynaptic CB1, while I-LTD in slices of rats subjected to withdrawal followed by repeated in vivo morphine exposure is dependent on both presynaptic CB1 and postsynaptic LTCC. Combined with previous findings from LTP (E-LTP) and LTD (E-LTD) of excitatory synaptic transmission in the hippocampus^13^,^14^,^30^, these results suggest that adaptations of the excitatory and inhibitory synapses in the hippocampus occur during opioid addiction and in turn may contribute to the storage of opioid addiction/reward-related memory and the persistence of opioid relapse.

### The hippocampus-associated pathological memory

Activity- or experience-dependent synaptic plasticity in the hippocampus has been proposed as the cellular substrate of information processing and memory formation in the brain under both physiological^10^,^11^ and pathological conditions, including addiction^12^,^13^,^14^,^30^. It has been well documented that drug exposure produces rewarding effects mediated by the mesolimbic dopamine system^31^,^32^, while withdrawal produces stress responses indicated by an increased level of corticosteroid in the hippocampus and VTA^15^,^31^,^32^. Thus, both hippocampal E-LTP and I-LTD are largely enhanced by acute withdrawal which could be a stressor^33^, and the lack of stress effect on impairing hippocampal E-LTP may also enable more long-lasting addiction memory. Furthermore, our previous study has shown that injection of the glucocorticoid receptor antagonist RU38486 into either the hippocampus or the NAc blocks the formation of opioid conditioned place preference^5^. Evidence from cocaine abuse suggests that cocaine-associated memory may be encoded by the hippocampal-NAc pathway, and thus electrical stimulation of the pathway may retrieve the pathological addiction memory and further triggers relapse^9^. It is therefore likely that opioid exposure and acute withdrawal may contribute to different aspects of pathological memory, marked by drug cues or stress-triggered relapse, i.e. persistence of addictive behaviors.

### Combinatorial plasticity of E-LTP and I-LTD in addiction memory

Repeated opioid self-administration increases presynaptic cannabinoid receptor 1 (CB1R) function in mesolimbic dopamine circuits including the hippocampus^34^. Evidence demonstrates that repeated opioid exposure blocks E-LTP^13^ of and facilitates E-LTD-like modification^12^,^30^, but opioid withdrawal facilitates E-LTP^14^ and blocks E-LTD-like modification^30^ in the hippocampus. The present findings showed that repeated opioid exposure blocks CB1-dependent I-LTD, but subsequent withdrawal facilitated I-LTD largely with CB1- and LTCC-dependent components. Thus, combinatorial plasticity in opioid addiction might show at least three aspects. Firstly, E-LTP-like modification occurs^12^ without affecting I-LTD (Fig. 1B) after single opioid exposure; both E-LTP^13^ and I-LTD (Fig. 1C) are blocked after repeated opioid exposure. Secondly, both E-LTP^14^ and I-LTD (Fig. 2C) are largely enhanced after acute withdrawal for 3-5 days. Finally, acute withdrawal largely enhances I-LTD which has both CB1R- and LTCC-mediated components.

The causality between E-LTP and I-LTD is documented in many excitatory synapses including in the hippocampus^16^,^35^,^36^, and they may work together in the hippocampus of the mesolimbic dopamine circuits, and lead to much larger response to repeated opioid exposure and acute withdrawal. Our previous reports suggest that combinatorial plasticity^37^ may endow the hippocampus to detect and store new information^38^ or opioid-associated experience or event^12^ effectively. This view may be further strengthened by the present findings. Perhaps, in the adaptations to repeated opioid exposure and acute withdrawal, the dynamics of combinatorial mechanism of synaptic plasticity such as E-LTP with E-LTD, E-LTP with I-LTD and CB1- with LTCC-dependent I-LTD may enable addiction memories to be more powerful and long-lasting.

In summary, these findings demonstrate for the first time that a combinatorial plasticity mechanism in the inhibitory synapses of the hippocampus occurs with opioid addiction, and in turn may contribute to the persistent aspects of opioid addiction.

## Methods

### Animals

Male Wistar rats (3-4 weeks) were obtained from Kunming Medical University Animal Care Centre and were maintained at Kunming Institute of Zoology Animal Care Centre in accordance with the guidelines set forth by Chinese Academy of Sciences Animal Care and Use Committee. Animals were housed in plastic cages in a temperature-controlled (21°C) colony room on a 12/12 h light/dark cycle. Food and water were available *ad libitum*. All efforts were made to minimize the number of animals used.

### Morphine Treatment

Rats aged 3-4 weeks were treated with a single dose (SM; 10 mg/kg, s.c.) or repeated morphine (RM; 10 mg/kg, s.c., twice per day at 12 h intervals for 12 d) similar to those described previously^12^,^39^,^40^. Some SM and RM rats were further subjected to withdrawal for 3-5 d (RMW) that caused obvious withdrawal signs such as grooming, diarrhea etc^14^,^15^. In control groups, rats were injected with sterile saline (saline; 0.6 ml/kg, i.p.). All experiment protocols were approved by Chinese Academy of Sciences Animal Care and Use Committee.

### Slice Preparation and Electrophysiology

Slices were prepared by using techniques similar to those described previously^41^,^42^. The rat brain was quickly removed and placed in ice-cold artificial cerebral spinal fluid (ACSF) in vibroslicer chamber. Coronary-sectioned 400-μm-thick hippocampal slices were cut and transferred into a submersion-type incubation chamber with 300 ml ACSF heated to 35 ± 1°C for 1 h recovery. ACSF contained (in mM): NaCl 120, KCl 2.5, NaHCO_3_ 26, NaH_2_PO_4_ 1.25, CaCl_2_ 2.0, MgSO_4_ 2.0 and D-glucose 10; and was saturated by continuous perfusion of gas mixture of 95% O_2_ and 5% CO_2_. Then, an incubated slice was gently transferred into a recording chamber, and held submerged between two nylon nets and maintained at room temperature (22-25°C). The recording chamber consisted of a circular well of low volume and was perfused constantly by ACSF at a flow rate of 4-5 ml/min. Recording chamber was placed on a stage of an upright Nikon microscope equipped with a 10 × objective and a 10 × ocular (600FN, Japan) to identify CA1 pyramidal neurons. Stimulating electrode was prepared by gluing together a pair of twisted Teflon-coated 90% platinum/10% iridium wires (50 μm inner diameter, 75 μm outer diameter, World Precision Instruments, Sarasota, Florida). Patch pipette was pulled from borosilicate glass tubing (1.5 mm outer diameter, 0.84 mm inner diameter, World Precision Instruments, Sarasota, Florida) with a Brown-Flaming micropipette puller (P-87; Sutter Instruments Company, USA).

Whole-cell inhibitory postsynaptic currents (IPSCs) were recorded at the holding potential of −70 mV in response to stimulation of the Schaffer collateral/commissural pathways. The whole-cell recordings were obtained by using recording electrode (3-6 M) containing pipette solution (in mM): CsCH_3_SO_3_ 100, MgCl_2_ 1, CsCl 60, HEPES 10, EGTA 0.2, Mg-ATP 2, Na-GTP 0.5, and QX-314 5, pH 7.2 (320 mosM). In order to block EPSCs, recording of IPSCs was performed in the continuous presence of the AMPA/Kainate and NMDA receptors antagonist kyneurenic acid (KYNA, 3 mM, dissolved in 0.1% NaOH) in bath solution. Baseline IPSCs were adjusted at a stimulus intensity to evoke about 50% of the maxim IPSCs amplitude. After a 10-min of stable baseline recordings, long-term depression of IPSCs (I-LTD) was induced by high-frequency stimulation (HFS) consisting of 2 trains (20s apart) each containing 100 pulses at 100 Hz, combined with depolarizing step of the postsynaptic neuron from −70 mV to 0 mV, similar to those described previously^24^. All recordings were made by using an Axopatch 200B amplifier. Signals were digitized at 20 kHz and filtered at 5 kHz and stored on computer. Series resistance was monitored by using a −5 mV, 50 ms pulse in each sweep of the IPSCs recording. Results from cells with more than 10% change in series resistance were excluded from analysis.

### Drug Application

All drugs were purchased from Sigma (St. Louis, Missouri). Drugs, except BAPTA, were applied in bath solution for 30 min before HFS and maintained unwashed at the following concentrations: cannabinoid receptor 1 (CB1) antagonist AM251 (2 μM), L-type calcium channel (LTCC) blocker lanthanum chloride (LaCl_3_; 20 μM). AM251 and LaCl_3 _were dissolved in ACSF. In experiments including postsynaptic calcium buffer, CsCH_3_SO_3_ (20 mM) were replaced by BAPTA (20 mM) in pipette solution.

### Data Analysis

Baseline was measured as the averaged IPSCs amplitude in 10-min recordings (20 sweeps). LTD was measured as the averaged IPSCs amplitude of the last 10-min recordings (20 sweeps). Results are reported as mean ± SEM% of baseline IPSCs amplitude. Each point in figures was average of two sweeps (1 min). Comparison between baseline and LTD was made by using Student’s *t*-test. Comparison among groups was conducted by using one-way ANOVA) followed by post hoc Turkey’s test. Significance level was set at *p* < 0.05.

## Supplementary Material

Supplementary Information Supplementary Information
