# Supplementary material for: Opioid addiction and withdrawal differentially drive long-term depression of inhibitory synaptic transmission in the hippocampus
Source: Sci Rep. 2015 May 5;5:9666. doi: 10.1038/srep09666 (PMC5386187; doi:10.1038/srep09666)
Supplement: Supplementary Information [file srep09666-s1.pdf]

Supplementary Information

Opioid addiction and withdrawal differentially drive long-tern depression of inhibitory synaptic transmission in the hippocampus

Huili Han[1,2,3], Zhifang Dong[1], Yunfang Jia[3,4], Rongrong Mao[3], Qixin Zhou[3] , Yuexiong Yang[3], Liping Wang[3], Lin Xu[3] and Jun Cao[3*]

[1]Chongqing Key Laboratory of Translational Medical Research in Cognitive Development and Learning and Memory Disorders，and Ministry of Education Key Laboratory of Child Development and Disorders, Children's Hospital of Chongqing Medical University, Chongqing 400014, China

[2]Center for Clinical Molecular Medicine, Children's Hospital of Chongqing Medical University, Chongqing 400014, China

[3]Key Laboratory of Animal Models and Human Disease Mechanisms, and KIZ/CUHK Joint Laboratory of Bioresources and Molecular Research in Common Disease, and Laboratory of Learning and Memory, Kunming Institute of Zoology, Chinese Academy of Sciences, Kunming 650223, China

[4]Kunming College of Life Science, University of Chinese Academy of Sciences, Beijing 100049, China

***Correspondence to:** juncao@vip.163.com

Supplementary methods

I-V curves

Whole cell voltage-clamp recordings were performed at room temperature at a holding potential of -70 mV. I-V curves were obtained by ramping the membrane potential from -70 mV to +70 mV by 10 mV interval.

Miniature IPSC recording

Miniature IPSCs (mIPSC) were recorded in whole-cell configuration. Series resistance and leak currents were monitored continuously during the experiments. Recordings started 5-10 min after a stable whole cell access was obtained. Recordings were terminated if series resistance and leak currents changed significantly.

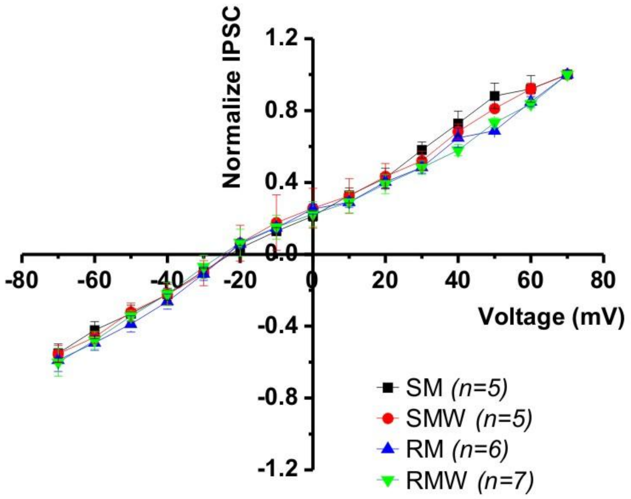

sFigure 1 The I-V relationship of IPSCs obtained from SM, RM and withdrawal groups. The holding potential was -70 mV and test potentials ranged from -70 mV to +70 mV in 10 mV increments. Reversal potential and current responses at various membrane potentials are not altered in all groups.

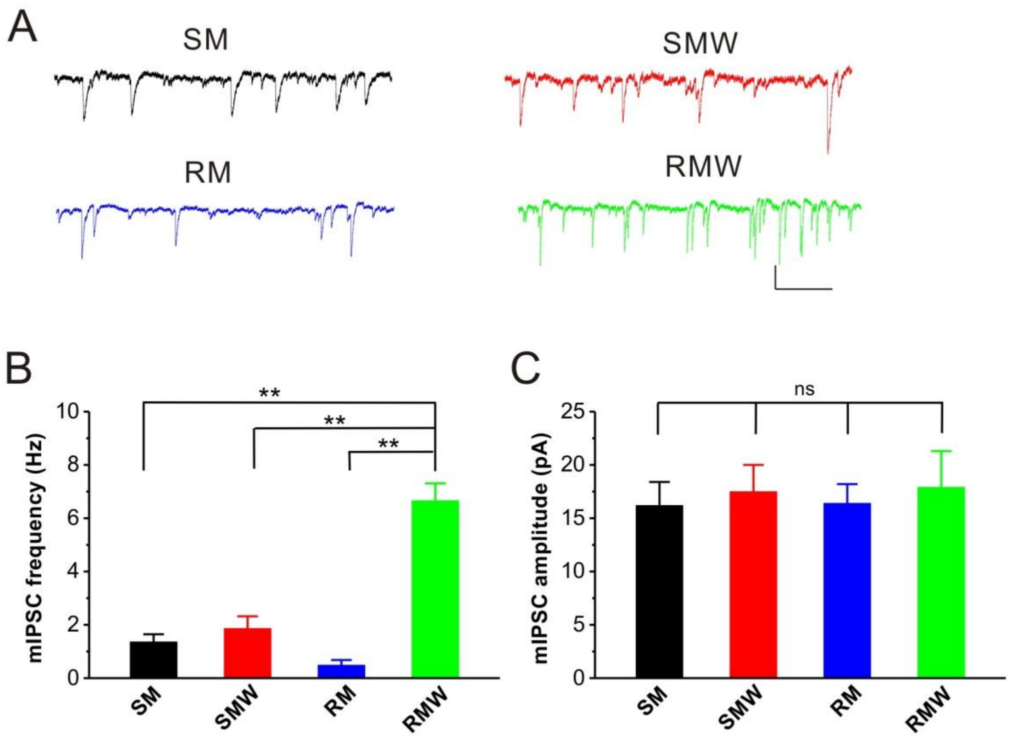

sFigure 2 The mIPSCs obtained from SM, RM and withdrawal groups. (A) Representative traces from corresponding hippocampal slices (Scale bar: horizontal = 1 s, vertical = 10 pA). **(B)** The mean frequency of sIPSCs was significantly increased in RMW group, compared with SM, SMW or RM groups. **p < 0.01, post hoc Turkey's test after ANOVA ($F_{(3, 28)} = 41.338$; $p < 0.001$). **(C)** The mean amplitude of mIPSCs remained unchanged among these groups. ns = no significantly difference, post hoc Turkey's test after ANOVA ($F_{(3, 28)} = 0.108$; $p = 0.954$).
